# Whisky, Wine and Beer

**Published:** 1909-03-06

**Authors:** 


### Whisky, Wine and Beer.

We note with some interest that the controversy concerning the deleterious effect of alcohol has recently been revived, and that attention has once more been drawn to the differences both in alcoholic strength and other constituents of the various alcoholic beverages. The extreme view taken by the exponents of anti-alcoholism is that alcohol in any form or quantity is, if not directly, at least remotely, injurious to health and deleterious to the human race. A less violent and probably wider spread thesis is that the injury to health produced by alcoholic beverages depends upon the quantity of the alcoholic content of the beverage, the associated constituents of the beverage, and the manner and form in which it is imbibed. Put in other words the latter view amounts to the assumption that there is a quantity of alcohol which may under certain conditions be taken without producing either physical or moral deterioration.

This is not the place to discuss the moral aspect of the question, nor does the elucidation of this problem essentially rest with the medical profession. Our function as doctors is to decide whether or not alcoholic beverages should be prescribed for individuals, and, if the answer be in the affirmative, above all what form and what dose of alcoholic beverage should be given. To tell a patient to take whisky, wine, or beer without telling him at the same time how much, and when and how often, is both neglectful and dangerous. Assuming some benefits are to be derived from alcohol, we can only expect them to accrue under specific circumstances, and of these specific circumstances clearly the most important are dose and form. In order to tell our patients we must know ourselves, and it is therefore incumbent upon every medical practitioner to know the alcoholic strengths and general secondary products of the ordinary alcoholic beverages, and at any rate not to prescribe an alcoholic beverage concerning the properties of which he is in ignorance. We have endeavoured on several occasions in these columns to consider the essential chemical and physiological properties of different alcoholic beverages, and have already published Reports upon Whisky and Wines, and shall shortly publish a Report upon Beer, including under this head lager beer and stout.

Science is measurement, and if the public could be made to grasp clearly the importance of accurately measuring their alcoholic beverages, not only would a great advance in the cause of temperance be made, but also an equal advance in therapeutics. We never dream of prescribing any medicine without clearly knowing what therapeutic effect we expect of it, and what dose in the given individual is *a priori* most likely to produce this effect. Surely this must be true of alcohol, and in prescribing any form of it let us first of all know what we want of it, and, secondly, how best to get the desired effect out of it. That alcohol does something, both teetotallers and drunkards are agreed, and any medical man who wants a desired effect in a patient can clearly judge by his clinical sense whether it is or is not obtained by a certain dose of alcohol. These considerations apply to the so-called dietetic as well as to the purely medicinal use of alcoholic beverages. In the former case, however, we must never lose sight of the fact that a certain quantity of fluid must be taken by the body during the day, and that the taste and flavour of this fluid may considerably influence the nutritive value which a given individual can extract from a given diet. There are certainly many people who take the lighter forms of alcoholic beverage, as, for instance, wine and beer, not because they want or they like the alcohol, but because they can digest and assimilate their food better with a drink of a specific flavour, only to be obtained as a by-product of alcoholic fermentation under certain conditions. In these cases the flavour is the essential active principle and the alcohol is the adjuvant.

The fact remains that we must drink something, and there is scarcely any beverage under the sun not open to some more or less grave exception. He only destroys who rebuilds, and those earnest but hypercritical philanthropists who are apt too readily to blame doctors for recommending this or that beverage should remember that the medical man has not only to tell a patient what he must not drink, but also what he must.

